# Assessing physical activity, mental health, and quality of life among older adults in Tehran, Iran: A cross-sectional study

**DOI:** 10.1371/journal.pone.0317337

**Published:** 2025-04-21

**Authors:** Mohammad VaezMousavi, Hadi Nobari, Amir Shams, Hamed Abbasi, Parvaneh Shamsipour-Dehkordi, Fariba Mohamadi, Andrew J. Brinkley, Mahdi Bayati, Lara Carneiro

**Affiliations:** 1 Imam Hossein University, Tehran, Iran; 2 LFE research group, Department of Health and Human Performance, Faculty of Physical Activity and Sport Science (INEF), Universidad Politécnica de Madrid, Madrid, Spain; 3 Department of Motor Behavior, Sport Sciences Research Institute, Tehran, Iran; 4 Department of Sports Medicine, Sport Sciences Research Institute, Tehran, Iran; 5 Department of Motor Behavior, Faculty of Sport Sciences, Alzahra University, Tehran, Iran; 6 Department of Sports Medicine, Sport Sciences Research Institute, Tehran, Iran; 7 School of Sport, Rehabilitation and Exercise Sciences, University of Essex, Colchester, United Kingdom; 8 Department of Exercise Physiology, Sport Sciences Research Institute, Tehran, Iran; 9 Physical Education Department, College of Education, United Arab Emirates University, Abu Dhabi, United Arab Emirates; Shahrood University of Medical Sciences, IRAN, ISLAMIC REPUBLIC OF

## Abstract

**Background:**

The World Health Organisation (WHO/EMRO) funded research aimed to evaluate the effects of physical activity (PA) levels and their relationship with mental health and quality of life (QoL) among older adults in Tehran, Iran.

**Methods:**

A cross-sectional design was used, and data from five different regions were collected from 7500 individuals aged over 60 years using four questionnaires: The International PA Questionnaire (IPAQ), Quality of Life Questionnaire (WHOQOL-26), General Health Questionnaire (GHQ-28), and a preliminary individual characteristics questionnaire. The IPAQ assessed PA, while the GHQ-28 evaluated mental health. The WHOQOL-26 assessed quality of life in four areas: physical health, psychological well-being, social relationships, and environment. Data analysis was conducted on MLwiN 3.05, whereby multilevel regression models were constructed.

**Results:**

Results indicated that most participants had low levels of PA, with 74.4% meeting low levels of PA. No meaningful variation in mental health or QoL was attributable to PA behavior across regions. However, age, marital status, dependents, and MET PA were found to significantly predict mental health scores, while MET PA, age, number of dependents, social welfare, gender, and education significantly predicted QoL values.

**Conclusion:**

The study highlights the need for executive support, coordination mechanisms, and appropriate infrastructures to improve the level of PA among Iranian older adults and promote mental well-being in this population.

## Introduction

According to United Nations reports, it has been predicted that the older adults population will increase from 600 million to two billion over the next 50 years [1]. This occurrence can be ascribed to advancements in science, health, society, and technology. However, although technological advances have provided many benefits to the community, it has also substantially reduced incidental physical activity (IPA) [2]. In the Middle East and North Africa (MENA) region, the past few decades have witnessed higher levels of urbanization, along with technological and transportation advancements. These shifts have contributed to a notable increase in sedentary lifestyles, and it is estimated that almost half of adults and three-quarters of young people/teenagers are insufficiently active to meet the recommended international guidelines for physical activity (PA) [3]. The most extensive national study on PA in Iran, examining the prevalence of IPA in the adult population, classified by demographics, geographical regions, and activity domains, revealed that the highest IPA prevalence was recorded in Bushehr, located in the southern part of the country, with a rate of 70.3%. Conversely, the lowest rates for both male and female IPA prevalence were noted in Zanjan, standing at 39.8% overall [4]. This increases the risk-ratio for non-communicable physical, psychological, and social illnesses [5]. Physical activity serves as a protective factor against non-communicable diseases, including cardiovascular disease, stroke, diabetes, and certain types of cancer [6]. Furthermore, engagement in PA is linked to enhanced mental health [7], delayed onset of dementia, and an improved quality of life (QoL) [8].

The assessment and recognition of PA levels among communities and different age groups are essential [9]. Considering the specific needs of older adults, QoL is one of the essential psychological variables related to health and well-being [10,11], and is a well-known basic index of physiological, functional, and psychological health [12]. Health-related QoL is defined by physical, psychological, social, and familial well-being [11,13]. It influences older adults’ feelings, expectations, experiences, and beliefs [14]. Researchers believe that QoL in older adults means having an independent life, well-being, and good daily function [13,14].

Older people with higher PA levels tend to have more favorable mental and general health [15,16]. As an illustration, Asztalos et al. established a notable positive correlation between elevated levels of PA and mental health across both genders [17]. In a broader investigation, Abu-Omar et al. explored the association between PA and mental health across 17 countries in the European Union, revealing a generally positive relationship. Nevertheless, this correlation was not evident in certain countries, including Greece, Austria, and Italy [18], suggesting that perhaps geographical, socioeconomic, and cultural factors may mediate such relationships, as shown elsewhere [19–21]. Complexity in determining the relationship between PA and health outcomes may arise from: (a) the absence of agreement on establishing optimal benchmarks for the intensity, type, frequency, and duration of PA to enhance health status [22–24], despite general recommendations being provided by recognized entities [25]; (b) individual differences in acute and chronic responses to PA programs [26–28]; and (c) different relationships between PA and mental health due to personal characteristics such as age, gender, genetic predispositions, baseline health status, psychological resilience, and lifestyle factors such as diet or sleep habits.

A Middle East study that was developed In Iran, which evaluated research investigating the impact of both low and moderate-intensity aerobic exercise on the psychological well-being (PWB) and QoL in older individuals reveals that aerobic exercise enhances both PWB and QoL. Notably, moderate-intensity exercise appears to yield greater benefits compared to low-intensity, establishing a positive dose-response relationship [29]. Hence, in this study, we explored whether moderate or high-intensity PA produces greater benefits than low-intensity on mental health and QoL in Iranian older adults.

Many studies have been undertaken in various nations over the last decade. Nonetheless, there is a pressing necessity to undertake this task in various countries, driven by differences in geographical location, socio-cultural context, and economic standing, aiming to enhance and update community awareness. As a result, the World Health Organization, as the world’s primary steward of health, encourages and supports researchers in various countries to pursue their goals [29]. With the assistance of the World Health Organization, this project was the first of its kind in the Middle East. The absence of any real data in this region with solid science that compares the different regions of Tehran (North, South, East, West and Center) means that this study can advance the knowledge of mental well-being in this specific context and population.

One study that assessed the spatial distribution of sports spaces within walking distance in Tehran demonstrated that, considering the population density, areas with the highest population and residential density exhibit the fewest facilities. Interestingly, regions boasting more sports spaces per capita seemed to have the least accessibility to sports facilities [30].

It has been observed that an increased number of sports spaces per capita does not necessarily translate to greater accessibility within walking distance. This indicates that major sports facilities are situated in areas with relatively limited opportunities for local utilization. Recent research has predominantly focused on community sports in metropolitan areas, particularly in Tehran. For instance, AllahMoradi et al. study, which formulated a model for community sports in Tehran’s metropolis, identified factors influencing community sports, including macro-policy, development-sector requirements, responsibilities of the organization overseeing community sports, sports city infrastructure, urban unit management, and the overall metropolitan environment [31].

The objective of this study was to explore the correlation between PA, QoL, and mental health in individuals aged over 60 residing in Tehran, Iran. Additionally, findings indicated that an increased availability of sports spaces per capita did not have a significant impact on the investigated factors. It was hypothesized that there would be a positive relationship between PA and QoL and that the relationship would be mediated by mental health. The study also aimed to investigate the association between PA and mental health in older adults. The findings from this research could offer valuable insights for both community members and sports officials.

## Materials and methods

### Study design

A cross-sectional research design was employed to address the study’s inquiries. Written permissions were secured from the original authors to utilize the International Physical Activity Questionnaire (IPAQ) [32–34], Quality of Life Questionnaire (WHOQOL-26) [35], and General Health Questionnaire (GHQ-28) [36]. Adhering to the International Quality of Life Assessment protocol [37,38], each instrument was translated into Persian, with efforts made to coordinate and unify the Persian and English versions. The questionnaires’ validity was ascertained, involving three raters for each region.

The IPAQ [32] was employed to assess PA in older adults, evaluating the intensity, duration, and frequency of PA conducted by each individual in the preceding week. Mental health was appraised using the GHQ-28, which encompasses four subscales addressing physical symptoms, anxiety, social dysfunction, and depression [39]. Subsequently, a preliminary individual characteristics questionnaire was developed based on the study’s needs and objectives. Preliminary testing involved administering the Persian version of the questionnaire to 30 individuals within a similar target group. Ten experienced researchers (i.e., minimum of a master’s degree), who were trained to interact with participants, administered questionnaires. All questionnaires were completed by participants alone or with the help of researchers.

All participants provided free informed consent before participation. The recruitment period for this study was between 23.09.2015 and 22.12.2015.

### Participants and sampling

The sample size for this study was determined using a well-established statistical approach designed for large population studies. Cochran’s sample size calculation method, which is commonly used in cross-sectional research, was utilized. This method considers key factors such as the desired confidence level, the expected prevalence of the outcome of interest, and the acceptable margin of error. For our study, we assumed a confidence level of 95%, meaning that we wanted to be 95% confident that our sample would accurately represent the population. To account for the variability in PA levels and mental health, we estimated a prevalence rate of 50%, which is a conservative choice that maximizes the required sample size when the actual prevalence is unknown. Additionally, we set a 5% margin of error to ensure a high level of precision in our estimates.

The research population was older adults (i.e., > 60 years of age) from Tehran, Iran. Given that, according to the latest census in 2011, the older adult population was 1,102,123 people, we calculated an initial sample size. However, since the population is finite, we adjusted the sample size using the finite population correction method. This adjustment ensures that the sample is not unnecessarily large while still being representative. After applying these calculations and considering potential non-response and incomplete data, we increased the sample size to 7,500 participants to ensure robustness and allow for meaningful subgroup analyses across different regions and demographic factors.

First, Tehran was geographically divided into five regions: North, South, East, West, and Center. A total of 1,500 older adults were selected from each region using a multi-stage random sampling procedure. From each of these regions, one district was randomly selected, and then three neighborhoods within each district were chosen at random. Subsequently, five parks were randomly selected from each neighborhood. From each park, 100 older adults were recruited, bringing the total sample to 7,500 participants. Written consent was obtained from all participants prior to their involvement in the study.

### Ethics approval and consent to participate

The study was approved by the Ethics Committee of the Sport Sciences Research Institute of Iran (SSRI.REC.1394.101). All participants voluntarily signed informed consent forms prior to taking part in the study. The research adhered to the principles outlined in the Declaration of Helsinki throughout its course.

### Measures

The IPAQ, or International Physical Activity Questionnaire, assesses various activities such as work-related tasks, movement habits, household chores, and leisure-time PA. It measures an individual’s PA over the preceding week, evaluating these activities based on metabolic equivalent (MET) units. The MET unit is a measure used to estimate energy expenditure during PA, with walking assigned 3.3 MET, moderate PA rated at 4 MET, and vigorous PA at 8 MET.

The questionnaire provides two ways to express an individual’s level of PA:

Firstly, the total weekly PA is quantified in MET minutes per week, combining walking (MET ×  minutes ×  day), moderate PA (MET ×  minutes ×  day), and vigorous PA (MET ×  minutes ×  day) throughout the week.
Secondly, the classification of PA is presented in three levels: low, moderate, and high.

High PA entails engaging in high-intensity activity for at least three days a week, accumulating a minimum of 1500 MET-minutes, or having a mix of high, moderate, and walking activities totalling at least 3000 MET-minutes over seven days or most days of the week. Moderate PA involves either 20 minutes of high-intensity activity on three or more days per week or engaging in intense, moderate, or walking activity for at least 30 minutes on five or more days per week. Low PA is characterized by either not reporting any activity or reporting activities that are neither high nor moderate.

The IPAQ’s validity and reliability have been established by Craig et al. in a study conducted across 12 countries [40]. They reported a Cronbach’s alpha of 0.80 for reliability and validity ranging from 0.64 to 0.70. Saglam et al., in another study, reported the reliability and validity of the questionnaire at 0.78 and 0.66, respectively. Using Cronbach’s alpha coefficient in this study, the questionnaire reliability was established at 0.83 [41].

The WHOQOL-26, a shortened version of the World Health Organization Quality of Life assessment, was employed to evaluate the participants’ QoL. Comprising 26 items across four components, the scale assesses physical, psychological, environmental, and social communication aspects. Trompenaars et al. demonstrated reliability and validity in older adults, with reported coefficients ranging between 0.66 and 0.80 [42]. Other studies by Skevington et al. [43] and Chien et al. [44] also affirmed high levels of reliability and validity. In our research, the questionnaire’s reliability, assessed using Cronbach’s alpha coefficient, ranged from 0.69 to 0.93.

The GHQ-28 was used to assess Geriatric Mental Health, covering four subscales: physical symptoms, anxiety, social dysfunction, and depression. Its reliability has been confirmed in previous studies [36], including validation with the Iranian population [45]. Notably, lower values on this questionnaire indicate better mental health, a crucial aspect for interpreting the study’s findings. In addition, demographic data, including age, sex, socioeconomic status, educational level, specific diseases, surgical history, marital status, and the number of children, were collected through a demographic questionnaire.

### Analysis

Data analysis was conducted on MLwiN 3.05, whereby multilevel regression models were constructed. Descriptive statistics and bivariant correlations (alpha set at *p* = .05) were calculated for all continuous variables. Following replacing outliers (i.e., 16 hours of PA per day) within MET minutes of PA per week with the nearest normally distributed value, data were normally distributed within acceptable ranges for skewedness (±3) and kurtosis (±10). Multilevel models are helpful in analyzing non-normally distributed equivocal data with heterogenous variance across people, places, and regions [46]. Multilevel regression models were constructed to explore the impact of participation in PA on the mental health and QoL of older adults residing within Teheran. Within each model, participants (Level 1) (n = 7500) were nested into their ‘park’ (e.g., local authority) (Level 2) (n = 15), which was nested into a region of the city (Level 3) (n = 15). The analysis sought to explore any interindividual variance in mental health and QoL as a function of participation in PA across regions. MET minutes of PA, gender (male vs. female), age, marital status (married/partnered vs. single/widowed), employment status (employed vs. unemployed), education (high school vs. degree plus), surgical history (yes vs. no), number of dependents, social welfare, and social-economic status (earnings) were entered as fixed predictors. Each model was estimated through Iterative Generalized least squares and constructed in four stages. Foremost, a variance component-only model was constructed to establish the intraclass correlation coefficient (ICC) on the park level (model 1). ICC values > .20 indicate a multilevel model’s need [47]. Fixed predictors (grand mean centered) were entered into a random intercept model (model 2). To explore variance explained on the regional level, random slopes were constructed by allowing health outcomes to vary as a function of change in PA participation (model 3). Level 1 interaction effects were tested between independent and dependent variables. At each stage, the model’s fit was assessed using both 2 * loglikelihood and χ^2^ distribution tests for significance.

## Results

### Descriptive statistics and bivariant correlations

Descriptive statistics and bivariant correlations are presented in Table 1. Data indicates that 64.6% of participants were prescribed at least one medication, 25.4% were prescribed two, 4.8% prescribed three, and 3% were prescribed four. Two point two percent were not prescribed medication. Most participants were not employed (65.4%). The remaining participants (34.6%) were employed within the education, health, or sales sectors or were self-employed. Furthermore, 16.57% were employed with a second job (Table 2). Activities of daily living (ADL) include personal hygiene, dressing, toileting, eating, mobility, and manual tasks, such as pouring a glass of water. Most participants (91.8%) were unable to complete every ADL independently. More specifically, 4.2% could complete five activities, 2.6% could complete four, 1.2% could complete three, and 0.2% could complete two without assistance. Stratified, the percentages of participants meeting low, moderate, and high levels of PA were 74.4%, 15.4%, and 10.2%, respectively. Social welfare is ranked in five levels. Social welfare and average earnings were ranked across regions of Teheran (1 = lowest welfare/earnings and 5 = highest welfare/earnings). Data indicate the North had the highest welfare standards/earnings, while the South reported the lowest welfare and earnings (Table 3).

**Table 1:** Demographic data.

Regions	Gender		Age (M ± SD)		Marital status			
	Men (N)	Women (N)	Men	Women	Married	Widow or widower	Divorced	Single
**North**	954	546	74.5 ± 5.5	73.2 ± 6.7	72% (n = 1080)	14% (n = 210)	12% (n = 180)	2% (n = 30)
**South**	840	660	73.1 ± 6.5	70.4 ± 6.1	70% (n = 1050)	17% (n = 255)	10% (n = 150)	3% (n = 45)
**West**	990	510	76.5 ± 5.5	69.5 ± 5.7	65% (n = 975)	20% (n = 300)	13% (n = 195)	2% (n = 30)
**East**	1002	498	70.5 ± 3.5	71.5 ± 4.3	73% (n = 1095)	17% (n = 255)	8% (n = 120)	2% (n = 30)
**Center**	880	620	72.3 ± 4	72.7 ± 3.7	70% (n = 1050)	22% (n = 330)	7% (n = 105)	1% (n = 15)
***Total***	**4666**	**2834**	**73.4 ± 5.3**	**71.5 ± 5.1**	**70% (n = 5250)**	**18% (n = 1350)**	**10% (n = 750)**	**2% (n = 150)**

N, number; M, Mean; SD, Standard Deviation.

**Table 2:** Physical activity, mental health and quality of life.

Regions	Physical activity			Mental health						Quality of life				
	Low	Moderate	High	Somatic symptoms	Anxiety and insomnia		Social dysfunction	Severe depression	GHQ-28 total scale	Physical health	Psychological health	Social relationships	Health environment	WHOQOL-26 total scale
**North**	63.0% (n = 945)	23.0% (n = 345)	14.0% (n = 210)	15.9 ± 5.4		15.6 ± 5.5	14.8 ± 5.6	16.5 ± 5.7	*62.8 ± 17.6*	10.3 ± 2.7	10.5 ± 2.6	10.4 ± 2.6	10.9 ± 2.6	*41.4 ± 8.0*
**South**	83.9% (n = 1259)	9.1% (n = 136)	7.0% (n = 105)	16.5 ± 5.1		15.9 ± 6.0	16.1 ± 5.6	16.2 ± 6.0	*64.6 ± 19.3*	9.9 ± 3.0	10.3 ± 2.6	10.1 ± 2.8	10.3 ± 2.7	*40.6 ± 9.0*
**West**	74.0% (n = 1110)	16.0% (n = 240)	10.0% (n = 150)	16.5 ± 4.9		16.3 ± 5.6	16.2 ± 5.5	16.1 ± 5.7	*65.1 ± 18.4*	10.5 ± 2.9	10.2 ± 2.8	10.4 ± 2.8	10.6 ± 3.3	*41.1 ± 10.1*
**East**	78.8% (n = 1182)	14.2% (n = 213)	7.0% (n = 105)	16.8 ± 4.5		16.6 ± 5.3	17.7 ± 5.5	17.3 ± 5.7	*67.3 ± 18.4*	9.7 ± 2.6	9.6 ± 2.5	9.6 ± 2.5	9.7 ± 2.8	*38.6 ± 7.9*
**Center**	72.4% (n = 1086)	14.6% (n = 219)	13% (n = 195)	17.1 ± 5.0		17.3 ± 5.1	17.7 ± 5.4	17.2 ± 5.4	*69.4 ± 17.1*	10.1 ± 2.8	10.0 ± 2.7	9.9 ± 2.5	10.1 ± 2.9	*40.1 ± 9.1*
***Total***	**74.4% (n = 5612)**	**15.4% (n = 1153)**	**10.2% (n = 735)**	**16.5 ± 5.0**		**16.4 ± 5.5**	**16.5 ± 5.6**	**16.7 ± 5.7**	**65.9 ± 18.3**	**10.1 ± 2.5**	**10.1 ± 2.7**	**10.1 ± 2.7**	**10.2 ± 2.9**	**40.5 ± 8.9**

GHQ-28, General health questionnaire; WHOQOL-26, World Health Organization Quality of Life Questionnaire.

**Table 3:** Descriptive statistics and bivariate correlations between variables.

Variable			M ± SD			*r(p)*											
		1	2	3	4	5	6	7	8	9	10	11	12	13	14	15	16
1. Age	72.51 ± 5.61	–															
2. Dependents (n)	1.54 ± 1.11	−.01	–														
3. MET’	741.61 ± 756.33	.04**	.04***	–													
4. Somatic symptoms	16.54 ± 5.00	−.05***	−.07***	−.55***	–												
5. Anxiety and insomnia	16.35 ± 5.51	−.07***	−.05***	−.60***	.50***	–											
6. Social dysfunction	16.47 ± 5.62	−.06***	−.02 *	−.57***	.50***	.60***	–										
7. Severe depression	6.65 ± 5.72	.05***	−.06***	−.60***	.53***	.66***	.68***	–									
8. Total (GHQ)	65.85 ± 18.30	.04***	−.07***	−.69***	.73***	.82***	.83***	.86***	–								
9. Physical health	10.08 ± 2.74	.05***	.05***	.41***	−.34***	−.36***	−.34***	−.34***	−.41***	–							
10. Psychological health	10.10 ± 2.65	.04***	.02 *	.45***	−36***	−.38***	−.38***	−.37***	−.43***	.47***	–						
11. Social relationships	10.06 ± 2.64	.05***	.04***	.43***	−.34***	−.37***	−.367***	−.35***	−.42***	.56***	.55***	–					
12. Health environment	10.21 ± 2.89	.05***	.00	.42***	−.35***	−.36***	−.34***	−.35***	−.42***	.60***	.61***	.51***	–				
13. Total (QoL)	40.46 ± 8.92	.06***	.04***	.53***	−.43***	−.45***	−.44***	−.44***	−.52***	.81***	.81***	.80***	.84***	–			
14. Air pollution		−.12**	18**−	−.04**	.06**	.07**	.14**	.03**	.09**	−.04**	−.03**	−.06**	−.04**	−.05**	–		
15. Sport clubs (number)		.15**	.14**	−.23**	.04**	−.07**	−.08**	−.17**	−.03**	−.11**	.02 *	.04**	.06**	.04**	−.69**	–	
16. Parks (number)		.09**	−.20**	.00	−.07**	−.09**	.15**	−.02 *	−.10 *	−.00	.02	.04**	.01	.02	−.72**	.90	–
17. Leisure clubs (number)		.08**	−.15**	−.00	−.06**	−.07**	−.12**	−.00	−.08**	−.00	−.00	.02 *	−.00	.00	−.75**	.86**	.91**

M, mean. SD: standard deviation; MET’, MET minutes of physical activity; GHQ, General Health Questionnaire; QoL, Quality of life. * *p* = .05. **p = .01. ***p = .001

### Does participation in PA influence mental health?

Reflecting the need for a multilevel model, the ICC of the variance-only model on the park level was high (0.77) [47]. The random-intercept model (model 2) explained the variance of 1% of the regional and 58% of the park levels. A random-intercept and slope model (model 3) was constructed where mental health at the regional level could vary as a function of participation in PA. This explained 1% of the variance at the regional level and 58% at the park level. Data indicates mental health scores were significantly predicted by age (*β* = -0.071, *p* = 0.004), being widowed, single or divorced (*β* = 1.23, *p* = 0.001), dependents (*β* = -1.12, *p* = 0.001), and MET PA (*β* = 0.016, *p* = 0.001). However, model 3 indicated that no meaningful mental health variation was accountable to PA behavior across regions. Model fit statistics and parameter estimates are available in Table 4.

**Table 4:** Multilevel regression model predicting mental health and wellbeing.

Variable	*Model*	ICC	-2ll (^df^*p*-value)	*v*_0*k*_	*v*_3*k*_	*u*_0*j*_	*u*_2*j*_	*e*_0*ijk*_	*β(*SE)	*p*-value
	*Null Model*	.77	64120.43	5.06	3.35	261.03	6.61	69.03	65.83(1.03)	**–**
	*Model 1*	**–**	59444.607^*df = 12*^*, p = .001*)	1.19	.83	97.67	4.46	70.45	**–**	**–**
	*Model 2*	–	59360.673 (^*df = 13*^*, p* = .001)	.42	.34	95.94	4.42	70.06	–	–
Intercept (constant)									65.82 (.03)	.001
MET’									−.01 (.00)	.001
Gender (Women)									−.02 (.30)	.468
Age									−.07 (.02)	.004
Marital Status (SWD)									1.23 (.31)	.001
Employment (Yes)									−.36 (.30)	.110
Education (degree+)									.29 (.46)	.265
Dependents									−1.12 (.13)	.001
Social Welfare									−1.52 (.11)	.001
SES									.00(.00)	.99

Note: ICC, interclass correlation; -2ll, -2 * loglikelihood (deviance in IGLS estimation); Regional level (*v*_0k_, intercept; *v*_3*k*,_ slope). Park level (*u*_0jk_, intercept; *u*_2*jk*,_ slope). Participant level (e_0*ijk*_, intercept). MET’, Metabolic minutes of physical activity; SWD, Single Widowed or Divorced; SES, Social Economic Status.

### Does participation in physical activity influence markers of QoL?

The ICC of the variance-only model on the park level was high (0.68) [47]. The random-intercept model (model 2) explained 1% of the regional and 58% of the park levels variance. The random-intercept and slope model (model 3) allowed the QoL to vary as a function of participation in PA across regions. This explained 1% of the variance at the regional level and 56% at the park level. However, model 3 indicated no meaningful variation in the QoL accountable to PA behavior across regions. Data likewise indicates QoL values were significantly predicted by MET PA (*β* = 0.006, *p* = 0.001), age (*β* = 0.03, *p* = 0.02), number of dependents (*β* = 0.27, *p* = 0.001), and social welfare (*β* = 0.20, *p* = 0.001). Moreover, QoL values were meaningfully greater in women (*β* = 0.02, *p* = 0.001) and lower in those who were degree-educated (*β* = -0.48, *p* = 0.030). Model fit statistics and parameter estimates are available in Table 5.

**Table 5:** Multilevel regression model predicting quality of life.

Variable	*Model*	ICC	-2ll (^df^*p*-value)	*v*_0*k*_	*v*_3*k*_	*u*_0*j*_	*u*_2*j*_	*e*_0*ijk*_	*β*(SE)	*p*-value
	*Null Model*	.77	53608.960	1.20	.79	55.22	1.79	23.79	40.49 (.50)	**–**
	*Model 1*	**–**	51386.064(^*df = 12*^*, p = .001*)	.15	.12	33.30	1.52	24.12	**–**	**–**
	*Model 2*	–	51181.024 (^*df = 13*^*, p* = .001)	.20	.15	31.38	1.50	24.28	–	–
Intercept (constant)									40.62 (.24)	.001
MET’									.006 (.001)	.001
Gender (Women)									.02 (.17)	.001
Age									.03 (.01)	.020
Marital Status (SWD)									.02 (.18)	.450
Employment (Yes)									.05 (.17)	.370
Education (degree+)									-.48 (.27)	.030
Dependents									.27 (.07)	.001
Social Welfare									.20 (.06)	.001
SES									.00 (.00)	.99

ICC, interclass correlation; -2ll, -2 * loglikelihood (deviance in IGLS estimation); Regional level (*v*_0k_, intercept; *v*_3*k*,_ slope). Park level (*u*_0jk_, intercept; *u*_2*jk*,_ slope). Participant level (e_0*ijk*_, intercept); MET’, Metabolic minutes of physical activity; SWD, Single Widowed or Divorced; SES, Social Economic Status.

## Discussion

This study aimed to determine the relationship between participation in PA, mental health and QoL among Iranian older adults. Overall, 7500 older adults were selected from the North, South, East, West, and Center of Tehran. Participants were categorized into one of three PA levels including low, moderate, or high using a single composite indicator: total weekly PA in Metabolic Equivalents (MET-minutes), referred to as total PA. The results showed significant regional differences in PA levels, with the lowest prevalence of moderate PA at 9.1% in the South and the highest at 23% in the North, followed by 16% in the West, 14.6% in the Center, and 14.2% in the East. The North also had the highest prevalence of high PA at 14%, followed by the Center at 13%, the West at 10%, and the East at 7%. Most of the sample reported low levels of PA (74.4%).

The significant regional difference in PA levels suggests that inactivity is largely influenced by community-specific factors, highlighting the need for policies promoting PA at the local level. Country-specific interventions should consider the sociocultural and environmental factors that affect PA. The low PA levels observed in this study may be due not only to environmental factors, such as a lack of sports facilities and institutional support, but also to inadequate infrastructure for PA.

These variations may be linked to sample characteristics like physical performance, disease prevalence by region, and mental status, highlighting socioeconomic, cultural, and geographical differences. Moreover, PA is influenced by a complex interplay of, intrapersonal (e.g., fear of falling, being overweight, or pre-existing medical conditions) [48] and interpersonal factors (e.g., lack of social support, peer influence, or familial encouragement) [49]. When interpreting the findings of this study, it is essential to consider these factors. Investing in comprehensive, community-based lifestyle interventions may help transform the environment to promote healthier living across the entire community.

This study identified sociodemographic factors influencing PA engagement, revealing that older adults, individuals with lower education levels, married individuals, and females are more likely to encounter barriers to PA. This highlights the need for tailored PA interventions to address the specific challenges faced by diverse demographic groups. Additionally, pre-existing medical conditions were noted as barriers; 64.6% of participants have been prescribed at least one medication. Comorbidities may limit functional capacity for PA, particularly in older adults, who often have reduced exercise capacity due to aging’s effects on neuromuscular structure and function [50].

The present study found that mental health scores were significantly predicted by age, widowhood, marital status (single or divorced), dependents, and MET PA. Additionally, negative correlations were observed between moderate and high levels of PA and the total mental health score; since higher GHQ-28 scores indicate poorer mental health, this suggests that moderate and high levels of PA are associated with better mental health. Notably, PA behavior across regions showed no significant variations in mental health following model 3.

According to scientific literature, there is consistent evidence that doing exercise or PA at moderate to high intensity is associated with longevity [51,52]. Documented evidence also demonstrated PA’s benefits in maintaining or improving several mental health conditions among older adults, namely reduced depressive symptoms [53], reduced risks of cognitive impairment, and dementia [54]. For example, a meta-analysis analyzed the effects of exercise on depression severity in older adults and found a small to moderate effect (standardized mean difference =  -0.34, 95% confidence interval -0.52 to -0.17) of exercise on depression [55]. Another meta-analysis, which analyzed only randomized controlled trials, advocated that exercise should be considered a standard component of managing depression in older adults [50].

However, caution is needed because one recent review found limited evidence that exercise consistently improves cognitive function in people with dementia due to high heterogeneity between trials analyzed [56]. To better understand this data, knowing what type of PA was performed would be necessary. The most extensive survey that analyzed PA profile in the Iranian population demonstrated that in all age groups, the largest domain of PA was work (53.7%), followed by transport (33.6%) and recreation (12.8%) [4]. Thus, only 12.8% performed PA as a recreational activity associated with pleasure and leisure. Engaging in recreational groups and socially supported PA is shown to deal with stress, anxiety and depression and as mitigating the symptoms of Alzheimer´s disease [57].

The initial exploration of dementia prevalence and its correlates among older adults in Iran was conducted through the National Elderly Health Survey. The findings revealed dementia rates of 3.7% among those aged 60-64 years, 6.2% in the 65-69 age group, 10.4% in the 70-74 age group, 14.4% in the 75-79 age group, and 13.0% in those aged 80 and above. Notably, West Azerbaijan reported the lowest, while North Khorasan had the highest age-sex adjusted prevalence rate of dementia [58]. A growing body of evidence aligns with the 2017 Lancet Commission on dementia prevention, intervention, and care, highlighting potentially modifiable risk factors for dementia. These factors include limited education, hypertension, hearing impairment, smoking, obesity, depression, physical inactivity, diabetes, and low social engagement [59].

We found a significant negative correlation between mental health and QoL across all regions of Tehran, indicating that better mental health is associated with improved QoL. However, the strength of these correlations varied significantly by region, suggesting that moderator variables may mediate these relationships. Quality of life in this demographic is influenced by three key elements: absence of disease, adaptation to life circumstances, and mental well-being [60]. For example, a study found that sleep quality mediated the relationship between depression and QoL among older adults, accounting for gender and health variations [61]. This highlights the importance of self-care practices, particularly regarding sleep quality, in the aging process. According to model 3, our study showed no significant variations in QoL related to PA behavior across regions. The final linear regression model identified the impact of each variable, revealing that high levels of PA account for 29.2% of the variance in QoL. However, further evidence is needed to clarify this relationship.

## Limitations

Physical activity levels alone may not fully explain the findings; the type of PA performed could provide further insights. Additionally, relying on self-reported data may have introduced bias in measuring PA. To better understand the associations of interest, it is crucial to consider missing measurements for related factors, such as comorbidities, dietary habits, sleep quality, obesity, and financial resources. A key limitation is the difficulty in establishing causal relationships from cross-sectional analyses.

## Conclusion

In older adults, increasing PA levels may contribute to improved mental health and perceived QoL. However, it is essential to take into account geographical, economic, and cultural factors to effectively strategize and promote increased PA in specific locations or societal groups. The significance of policies aimed at promoting PA is increasingly acknowledged, highlighting the need for changes in public policy and the identification of existing barriers. The findings from this study could provide valuable insights for policymakers in choosing appropriate PA initiatives, as well as for researchers seeking to fill gaps in the current evidence base.

## Supporting information

S1 Appendix Appendix. (DOCX)

S1 File Data. (XLSX)

## Abbreviations

ADL: Activities of Daily Living
GHQ: General Health Questionnaire
ICC: Intraclass Correlation Coefficient
IPA: Incidental Physical Activity
IPAQ: International PA Questionnaire
MET: Metabolic Equivalent
PA: Physical Activity
QoL: Quality of Life
WHOQOL: World Health Organization Quality of Life Questionnaire.
