# Supplementary material for: Assessing physical activity, mental health, and quality of life among older adults in Tehran, Iran: A cross-sectional study
Source: PLoS One. 2025 Apr 21;20(4):e0317337. doi: 10.1371/journal.pone.0317337 (PMC12011300; doi:10.1371/journal.pone.0317337)
Supplement: S1 Appendix — (DOCX) [file pone.0317337.s001.docx]

**Supporting Information/Appendix files**

***Level of physical activity***

Physical activity level is a numerical representation of an individual's daily physical activity, serving as an estimate of their total energy expenditure [1, 2]. When combined with the basal metabolic rate, it helps calculate the necessary food energy intake to sustain a specific lifestyle. Various instruments, both direct and indirect, are available for measuring the level of physical activity. In this study, the International Physical Activity Questionnaire (IPAQ) was employed to evaluate the physical activity level among older adults.

***Mental health***

Mental health, as defined by the World Health Organization (WHO), encompasses "subjective well-being, perceived self-efficacy, autonomy, competence, inter-generational dependence, and self-actualization of one's intellectual and emotional potential, among others” [3]. For this research, mental health was operationally defined based on scores obtained from the General Health Questionnaire-28 (GHQ-28) [4].

***Quality of life (QoL)***

Quality of life (QoL) represents the overall well-being of individuals and societies, extending across various contexts such as international development, healthcare, politics, and employment [5, 6]. The short version of the WHOQOL-26 was utilized in this study to assess and characterize the quality of life [7] [7].

1. Mummery K, Schofield G, Caperchione C. Physical activity dose-response effects on mental health status in older adults. Australian and New Zealand journal of public health. 2004;28(2):188-92. Epub 2004/07/06. doi: 10.1111/j.1467-842x.2004.tb00934.x. PubMed PMID: 15233360.

2. Craig CL, Marshall AL, SjÖStrÖM M, Bauman AE, Booth ML, Ainsworth BE, et al. International Physical Activity Questionnaire: 12-Country Reliability and Validity. Medicine & Science in Sports & Exercise. 2003;35(8).

3. Trompenaars FJ, Masthoff ED, Van Heck GL, Hodiamont PP, De Vries J. Content validity, construct validity, and reliability of the WHOQOL-Bref in a population of Dutch adult psychiatric outpatients. Quality of Life Research. 2005;14(1):151-60.

4. Goldberg DP, Hillier VF. A scaled version of the General Health Questionnaire. Psychol Med. 1979;9(1):139-45. Epub 1979/02/01. doi: 10.1017/s0033291700021644. PubMed PMID: 424481.

5. Skevington SM, Lotfy M, O'Connell KA. The World Health Organization's WHOQOL-BREF quality of life assessment: psychometric properties and results of the international field trial. A report from the WHOQOL group. Quality of life Research. 2004;13(2):299-310.

6. Chien C-W, Wang J-D, Yao G, Sheu C-F, Hsieh C-L. Development and validation of a WHOQOL-BREF Taiwanese audio player-assisted interview version for the elderly who use a spoken dialect. Quality of Life Research. 2007;16(8):1375-81.

7. WHO. Development of the World Health Organization WHOQOL-BREF quality of life assessment. The WHOQOL Group. Psychol Med. 1998;28(3):551-8. Epub 1998/06/17. doi: 10.1017/s0033291798006667. PubMed PMID: 9626712.
